# Artificial Intelligence-Based Predictive Modeling for Early Detection of Sepsis in Hospitalized Patients: A Systematic Review and Meta-Analysis

**DOI:** 10.1097/CCE.0000000000001360

**Published:** 2025-12-05

**Authors:** Ghulam Husain Abbas, Palash Sen, Oviya Anjali Giri, Nawaid Hussain Khan

**Affiliations:** 1 Faculty of Medicine, Ala-Too International University, Bishkek, Kyrgyz Republic.; 2 Department of Medicine, Hinduhridaysamrat Balasaheb Thackeray Medical College and Dr. RN Cooper Hospital, Mumbai, India.; 3 Department of Surgery, Mayo Clinic, Rochester, MN.

**Keywords:** artificial intelligence, early detection, hospitalized patients, machine learning, predictive modeling, sepsis

## Abstract

**OBJECTIVES:**

This systematic review evaluates artificial intelligence (AI)-based predictive models developed for early sepsis detection in adult hospitalized patients. It explores model types, input features, validation strategies, performance metrics, clinical integration, and implementation challenges.

**DATA SOURCES:**

A systematic search was conducted across PubMed, Scopus, Web of Science, Google Scholar, and CENTRAL for studies published between January 2015 and March 2025.

**STUDY SELECTION:**

Eligible studies included those developing or validating AI models for adult inpatient sepsis prediction using electronic health record data and reporting at least one performance metric (area under the curve [AUC], sensitivity, specificity, or F1 score). Studies focusing on pediatric populations, lacking quantitative evaluation, or unpublished in peer-reviewed journals were excluded.

**DATA EXTRACTION:**

Data extraction followed preferred reporting items for systematic reviews and meta-analyses guidelines. Extracted variables included study design, patient population, model type, input features, validation approach, and performance outcomes.

**DATA SYNTHESIS:**

A total of 52 studies met the inclusion criteria. Most used retrospective designs, with limited prospective or real-time clinical validation. Commonly used algorithms included random forests, neural networks, support vector machines, and deep learning architectures (long short-term memory, convolutional neural network). Input data varied from structured sources (vital signs, laboratory values, demographics) to unstructured clinical notes processed via natural language processing. Reported AUC values ranged from 0.79 to 0.96, indicating strong predictive performance across models.

**CONCLUSIONS:**

AI models demonstrate significant promise for early sepsis detection, outperforming conventional scoring systems in many cases. However, generalizability, interpretability, and clinical implementation remain major challenges. Future research should emphasize externally validated, explainable, and scalable AI solutions integrated into real-time clinical workflows.

**KEY POINTS**

**Question**: Can artificial intelligence (AI) models using electronic health record data reliably detect sepsis earlier than conventional clinical scoring systems?

**Findings:** Across 52 studies, AI-based predictive models, especially those using machine learning and deep learning, achieved strong discrimination (AUC 0.79–0.96) for early sepsis detection, often outperforming traditional tools such as systemic inflammatory response syndrome and quick SOFA (qSOFA). However, external validation and real-time implementation remain limited.

**Meaning**: AI models hold substantial promise for earlier, more accurate sepsis recognition, but widespread clinical adoption will require improved generalizability, transparency, and workflow integration.

Sepsis—defined by Sepsis-3 as life-threatening organ dysfunction from a dysregulated host response to infection—remains a major global challenge (1). Recent analyses estimate 48–50 million cases and ≈11 million deaths yearly (1), about 20% of worldwide mortality (2). Low- and middle-income countries bear the greatest burden (1); children under five account for roughly 20 million cases annually (2). In high-income nations, sepsis is among the most expensive hospital conditions, costing greater than $20 billion per year in the United States alone (1, 2), with average per-patient costs greater than $30,000 (1). Thus, sepsis imposes immense clinical and economic strain worldwide.

Early recognition is vital, as each hour of treatment delay worsens outcomes (3). Yet early diagnosis is difficult because initial signs are nonspecific (3). Common scoring tools—systemic inflammatory response syndrome (SIRS), modified early warning score (MEWS), Sequential Organ Failure Assessment (SOFA), qSOFA—help flag risk but cannot reliably identify sepsis early (3, 4). SIRS favors sensitivity; qSOFA favors specificity (4). Consequently, sepsis is often detected only after organ dysfunction appears. Despite awareness campaigns and early-bundle protocols, diagnosis frequently lags. Traditional biomarkers and imaging remain inadequate (3–5). Hence, better predictive tools are urgently needed.

Artificial intelligence (AI) and machine learning (ML) can exploit high-dimensional electronic health record (EHR) data to uncover subtle physiologic patterns preceding sepsis (3). Algorithms analyzing vitals, laboratories, and demographics can forecast deterioration hours before clinical recognition (3–6). Methods include tree-based models, recurrent and convolutional neural networks, and ensemble systems. Experimental studies already show strong predictive accuracy.

However, challenges persist: generalization across settings, interpretability for clinician trust, data completeness, and ethical or regulatory oversight (7). Few models have undergone prospective validation. This systematic review therefore synthesizes the current state of AI-driven models for early sepsis detection (7). We evaluate ML approaches, predictive features, validation strategies, and clinical translation gaps to guide future development (6). Key study characteristics are summarized in **Supplemental Table 1** (https://links.lww.com/CCX/B588).

## METHODS AND MATERIALS

### Search Strategy

**Figure 1.**
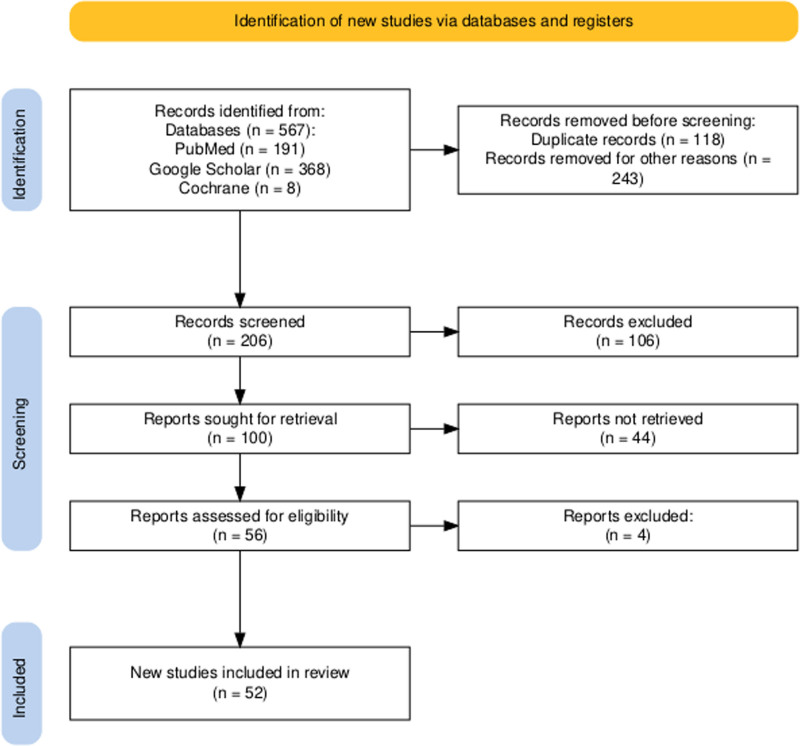
Preferred reporting items for systematic reviews and meta-analyses flowchart depicting the study selection process for the systematic review. The diagram outlines the number of records identified through database searches, screened for eligibility, excluded with reasons, and ultimately included in the final analysis. This provides transparency in the literature review methodology and ensures reproducibility.

We searched PubMed, Scopus, Web of Science, Google Scholar, and CENTRAL for studies (January 2015–2025) on AI/ML models for early sepsis detection. Controlled vocabulary and free-text terms combined “sepsis,” “early detection,” “machine learning,” and “electronic health records,” using Boolean operators and truncation. Search strings were refined for balance between recall and precision and limited to English-language journal articles. The process followed preferred reporting items for systematic reviews and meta-analyses (PRISMA) guidelines, with the flow diagram shown in **Figure 1**.

### Eligibility Criteria

Included studies met these conditions: 1) peer-reviewed original research, 2) adult inpatients (18 yr old or older), 3) AI/ML model for early sepsis detection or prediction, 4) EHR-based structured or unstructured data, and 5) greater than or equal to one quantitative performance metric. We excluded pediatric or neonatal work, non-peer-reviewed sources, prognostic or late-recognition models, and papers lacking methodological or quantitative details. These criteria narrowed the review to rigorous, directly comparable studies.

### Study Selection

Two reviewers (G.H.A. and P.S.) screened all records independently. Irrelevant titles or abstracts were removed, and remaining articles underwent full-text review. Disagreements were resolved by consensus or a third reviewer. Reasons for exclusion (e.g., wrong population or missing metrics) were documented in the PRISMA flowchart. Reference lists of included papers were hand-searched for additional studies.

### Data Extraction

Each eligible study was summarized using a standardized form capturing authorship, year, country, clinical setting, design, sample size, sepsis definition, algorithm type, input features, validation method, and performance metrics (area under the curve [AUC], sensitivity, specificity, accuracy, F1, etc.). Implementation attempts or usability evaluations were also noted. Two reviewers extracted data independently and cross-checked for consistency, resolving discrepancies by consensus.

### Quality Assessment

Because of methodological heterogeneity, quantitative meta-analysis was inappropriate (6). A narrative synthesis was used instead. We evaluated rigor and bias using an adapted prediction model risk of bias assessment tool framework (7) across four domains: participant selection, predictor measurement, outcome definition, and analysis. Calibration, decision-curve analysis, and transparency were noted when available. These assessments guided our qualitative comparison of strengths and limitations among studies.

## RESULTS

### Study Characteristics

Fifty-two studies met inclusion criteria, published 2015–2025 across Asia, North America, and Europe, with scarce data from Africa or South America. About 40% came from Asia (mainly China), 40% from North America, and the rest from Europe and elsewhere. Most were retrospective cohort analyses using EHR data (6); ≈ five were prospective or interventional trials. Sample sizes ranged from hundreds to millions of records (e.g., Steinbach et al [8] analyzed 1.38 million complete blood counts [CBCs] with > 2000 sepsis events). Typical cohorts included middle-aged to elderly adults with slight male predominance. Sepsis definitions varied (Sepsis-3, Sepsis-2, or *International Classification of Diseases* [ICD] coding), adding heterogeneity. Most models were developed in ICU settings within tertiary centers in Asia and North America. Performance metrics for each study appear in **Supplemental Table 2** (https://links.lww.com/CCX/B588), and **Supplemental Figure 1** (https://links.lww.com/CCX/B588) depicts AI-predicted risk vs observed outcomes.

### AI Methodologies

A broad range of algorithms was applied. Tree-based models dominated—Random Forest in ~14 studies and Gradient Boosting (XGBoost, LightGBM) in ~10—owing to robustness and feature-ranking ability. Deep neural networks appeared in ~8 studies, mainly recurrent (long short-term memory [LSTM]) or convolutional (convolutional neural network) architectures for time-series data. Support Vector Machines featured in early pilot work, later supplanted by ensemble and deep models. Ensemble techniques (stacking, bagging) combined multiple learners for marginal gains. Additional approaches included autoencoders, semi-supervised and transfer learning, and limited natural language processing (NLP) for unstructured text. Studies incorporating clinical notes or radiology reports reported modest performance improvement. A few explored novel inputs—waveform or continuous vital-sign streams—but most relied on structured EHR data. Simpler algorithms served as baselines in smaller datasets, whereas complex deep or ensemble models dominated large-scale work. As Chen et al (9) stated, “Logistic regression, SVMs, random forests, and gradient boosting … use vital signs, laboratory results, and clinical factors to predict the likelihood of sepsis” (8).

### Input Features

Core predictor categories were consistent across studies:

• Vital signs—heart rate, respiratory rate, blood pressure, temperature, oxygen saturation.
• Laboratory values—WBC, lactate, creatinine, blood urea nitrogen, platelet count, electrolytes, C-reactive protein, etc. Creatinine and lactate were frequent key markers.
• Demographics/comorbidities—age, sex, body mass index, chronic diseases.
• Medications/interventions—antibiotic administration, vasopressors, ventilation, fluid therapy.
• Unstructured data (NLP)—~10% of studies mined clinician notes or imaging text for sepsis-related terms.
• Novel features—electrocardiogram-derived metrics, ultrasound findings, time-trend derivatives, or multimodal fusions.

Most algorithms required 5–20 routine inputs; deep models could ingest hundreds. As Chen et al noted, such models rely on “vital signs … laboratory results … and clinical factors” (7). Heterogeneous EHR formats and coding practices hinder direct cross-site application (10).

### Model Performance

Reported performance was generally strong. Across the 52 studies, AUC-receiver operating characteristic ranged 0.79–0.96 (median ≈ 0.88), with sensitivities and specificities typically 0.80–0.95 (10). Many models outperformed traditional scores: an ensemble achieved AUC ≈ 0.93 vs. 0.64 for qSOFA and 0.69 for MEWS (6); Delahanty et al reported AUC 0.86 vs. 0.63 (SIRS) and 0.71 (qSOFA) (11). High performers included Kim et al (12) (2020, AUC 0.96), Wang et al (2021, 0.93), and Steinbach et al (2024, 0.872 internal; 0.805–0.845 external).

Comparisons remain difficult due to differing definitions and horizons (13). Calibration and decision-curve analyses were rarely provided (14). Still, evidence shows ML models outperform rule-based scores (15). Only ~40% included external validation, where performance dropped 5–10 points (8, 16). Validation and generalizability details appear in **Supplemental Table 3** (https://links.lww.com/CCX/B588).

### Implementation and Clinical Integration

Few systems reached deployment. Roughly five studies described live clinical use (17). Duke University’s “Sepsis Watch” and Dascena’s “InSight” were among the few tested in hospitals (3, 18). Integration hurdles included EHR connectivity, alert fatigue, and clinician acceptance (19). A review by van der Vegt et al (10) highlighted common barriers: data heterogeneity, infrastructure demands, privacy regulations, and insufficient user training (20). Excessive false positives reduced trust (21). Transparent reasoning and feedback mechanisms improved acceptance. Overall, pilot implementations proved feasibility but underscored the need for workflow adaptation and multidisciplinary support (22, 23).

## DISCUSSION

AI-driven models for early sepsis prediction achieve accuracy far above conventional scores, with many reporting AUC greater than 0.85 (6, 8). For example, one ML model achieved AUC 0.93, whereas qSOFA was 0.64 on the same cohort (6). By detecting deterioration hours earlier, such tools could expedite antibiotics and resuscitation, improving survival (3).

A major advantage is processing high-dimensional, longitudinal data (24–27). LSTM networks, for instance, capture evolving trends—rising lactate or falling pressure—that static rules miss (28, 29). Deep learning can identify new latent features (30, 31), explaining its superior sensitivity/specificity (> 85%).

Still, limitations remain.

Generalizability is the foremost concern (32). Many models were single-center, risking overfitting (33, 34). CBC model by Steinbach et al (8) dropped from AUC 0.872 to 0.805–0.845 externally (8). Differences in populations, coding, and labeling (Sepsis-2 vs. 3, ICD vs. clinical) impede transfer (35–38). Only ~40% used external validation (39, 40); multicenter collaborations are essential for robust generalization.

Explainability is another barrier. Complex neural and ensemble systems act as black boxes (41, 42). Clinicians hesitate to act on opaque alerts. Interpretability methods—SHapley additive explanations (SHAP), local interpretable model-agnostic explanations, attention heatmaps—can expose feature influence (e.g., elevated lactate + WBC = high risk) (43–45). Yet few studies included such analyses (46, 47). Regulatory bodies increasingly demand transparency (10, 11, 47, 48).

Workflow integration also limits impact. Even accurate tools fail if poorly embedded (49, 50). Real-time data streaming, alert presentation, and clinician engagement require technical and human-factor design. Alarm fatigue and liability concerns persist. Usability training and co-design with end-users improve adoption.

**Figure 2.**
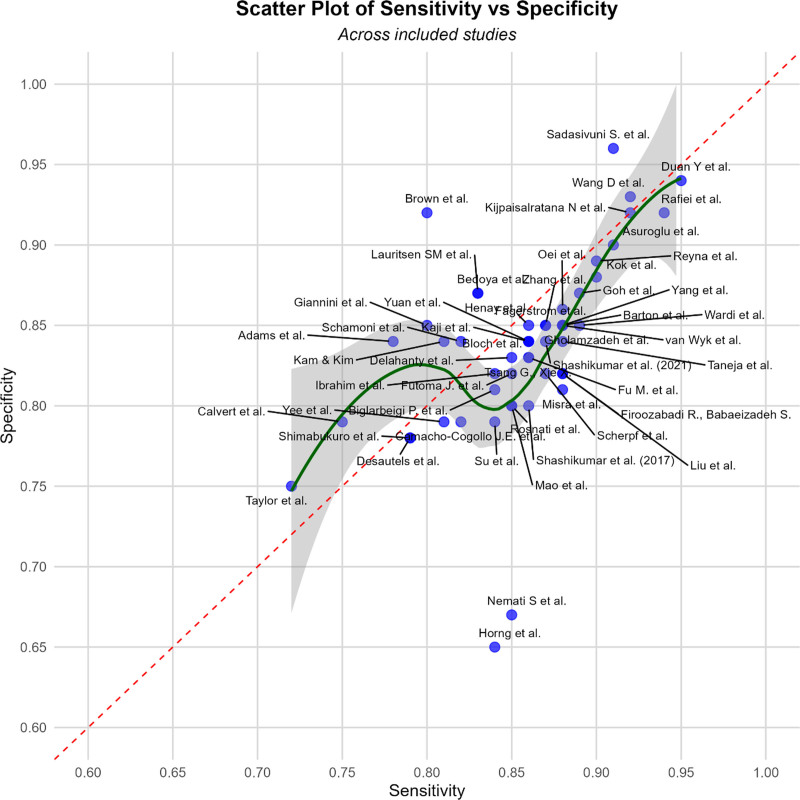
A scatter plot of sensitivity vs. specificity illustrating the architecture and operational steps of artificial intelligence (AI) systems used for early sepsis detection. The diagram includes components such as data input from electronic health records, preprocessing algorithms, model training and inference layers, risk scoring mechanisms, and clinical decision support outputs. This visualization emphasizes the end-to-end integration of AI in clinical practice across included studies.

Regulatory and ethical issues add complexity. Historical EHR data may encode demographic biases (51); without auditing, algorithms could amplify inequities. Privacy is critical (52). Federated or privacy-preserving methods offer mitigation. Legal accountability for missed or false alerts remains undefined (53). The draft guidance of Food and Drug Administration on AI/ML Software-as-a-Medical-Device emphasizes “good machine-learning practice,” lifecycle testing, and revalidation (13). Comparable frameworks are emerging internationally (54). **Figure 2** illustrates sensitivity vs. specificity among architectures.

In summary, AI sepsis prediction shows high accuracy but faces translational barriers in validation, interpretability, workflow, and governance (55). Addressing these is vital for real-world benefit.

## FUTURE DIRECTIONS

Key priorities include:

• Prospective multicenter validation: Most evidence is retrospective; randomized trials must evaluate real-time alerts and patient outcomes (56–58). The van der Vegt framework (10) offers deployment guidance.
• Pediatric and special populations: Sepsis also devastates children and pregnant or immunocompromised patients; tailored models are needed (59).
• Low- and middle-income countries: Given their disproportionate burden (2), LMIC-based models using simplified variables are essential (60, 61).
• Federated and privacy-preserving learning: Federated training enables multi-site modeling without sharing raw data (14). Early “FedSepsis” studies show comparable accuracy while preserving privacy (62, 63).
• Explainable AI (XAI): Future models should integrate interpretability—feature trajectories, risk rationales, SHAP or GAM-based transparency—to foster trust (64–66).
• Clinical trials and implementation science: Randomized trials must test outcome impact (66–68). Continuous-learning systems should be evaluated for safety over time.
• Regulatory pathways and standards: Alignment with emerging Food and Drug Administration and global AI/ML guidelines is required; open code and documentation promote reproducibility (59).

Interdisciplinary collaboration among clinicians, data scientists, engineers, ethicists, and regulators will be crucial to translate technical advances into equitable care.

## CONCLUSIONS

Artificial intelligence offers powerful tools for early sepsis detection. Our systematic review found that ML models using routine EHR data can forecast onset hours in advance with accuracy surpassing conventional scores. By enabling earlier intervention, AI could transform sepsis management. Yet widespread adoption demands validated generalization, interpretable reasoning, seamless workflow fit, and regulatory compliance. Progress depends on rigorous prospective trials and close collaboration between clinicians and technologists. With sustained, multidisciplinary effort, AI-driven prediction could become a reliable instrument to reduce sepsis mortality worldwide.

## Supplementary Material


