# Supplementary material for: Artificial Intelligence-Based Predictive Modeling for Early Detection of Sepsis in Hospitalized Patients: A Systematic Review and Meta-Analysis
Source: Crit Care Explor. 2025 Dec 5;7(12):e1360. doi: 10.1097/CCE.0000000000001360 (PMC12685403; doi:10.1097/CCE.0000000000001360)
Supplement: Supplementary file 1 [file cc9-7-e1360-s001.pdf]

Title Page:

Title: AI-Based Predictive Modeling for Early Detection of Sepsis in Hospitalized Patients: A Systematic Review and Meta-Analysis

Author(s):

Ghulam Husain Abbas[1]; Palash Sen[2], Oviya Anjali Giri[3], Nawaid Hussain Khan[1]

Affiliations:

1. Faculty of Medicine, Ala-Too International University, Bishkek, Kyrgyz Republic.
2. Hinduhridaysamrat Balasaheb Thackeray Medical College and Dr. RN Cooper Hospital, Mumbai, India
3. Department of Surgery, Mayo Clinic, Rochester, MN, USA

Corresponding author:

Ghulam Husain Abbas, Faculty of Medicine, Ala-Too International University, Bishkek, Kyrgyz Republic.

Email: ghulamhusainabbas.abbas@alatoo.edu.kg

Word count abstract: 262 words

Word count manuscript (without references): 2054 words

Running title: AI-Based Predictive Modeling

Type of Manuscript: Systematic Review

Conflict of Interest: None

Keywords: Sepsis, Artificial Intelligence, Machine Learning, Early Detection, Predictive Modeling, Hospitalized Patients

Table of Contents:

Supplemental Table 1 - Study Characteristics..Page 3

Supplemental Table 2 - Performance Metrics..Page 9

Supplemental Table 3 - Model Validation and Generalizability........................Page 12

Supplemental Figure 1 ………………………………..........................Page 14

Supplemental Table 1 - Study Characteristics

Summary of key features across 52 included studies evaluating AI applications in early sepsis detection. The table includes information on the first author, publication year, study location, clinical setting, design, sample size, patient population, and sepsis definition used. This provides context for the heterogeneity of methods, populations, and diagnostic criteria across studies.

Serial Number	Author (First Author et al.)	Year	Country	Setting	Study Design	Sample Size	Population Characteristics	Sepsis Definition
1	Lauritsen SM et al.	2020	Denmark	Hospitals (retrospective)	Retrospective multi-center	69,423 ICU admissions (3,173 sepsis)	Adult patients (Danish EHR data)	Sepsis-2 (2001 criteria)
2	Wang D et al.	2021	China	ICU (hospital)	Retrospective cohort	4,449 infected ICU patients	Adult ICU patients with infection	epsis-3 (Sepsis-3 criteria
3	Kijpaisalratana N et al.	2022	Thailand	ED (single center)	Retrospective cohort	133,707 ED visits (mixed patients)	Adult ED patients presenting with infection	al diagnosis (clinician-de
4	Nemati S et al.	2018	USA	ICU (single center)	Retrospective cohort	27,527 (8.6% developed sepsis)	Adult ICU patients (Emory)	Sepsis-3 (SOFA-based)

5	Duan Y et al.	2023	China	ICU (single center)	Retrospective cohort	282 patients	ICU patients with infection (Shanghai)	Sepsis-2 (SIRS-based)
6	Rosnati et al.	2021	Netherlands	ICU (MIMIC-III)	Retrospective	22,007	Adult ICU patients (vitals, labs)	Sepsis-3
7	Zhang et al.	2021	China	Emergency Department (DII Challenge data)	Retrospective	178,843	ED patients with EHR data (demographics, labs)	Sepsis-2
8	Shashikumar et al.	2021	USA	ICU (Emory, UCSD, MIMIC-III)	Retrospective	85,046	ICU patients (vitals, labs, demographics)	Sepsis-3
9	Aşuroğlu et al.	2021	Turkey	ICU (MIMIC-III)	Retrospective	5,154	ICU patients (vitals)	Sepsis-3
10	Oei et al.	2021	Netherlands	ICU (MIMIC-III)	Retrospective	48,632	ICU patients (labs, vitals)	Sepsis-3
11	Rafiei et al.	2021	China	ICU (PhysioNet/CinC Challenge)	Retrospective	40,336	ICU patients (demographics, labs, vitals)	Sepsis-3
12	Goh et al.	2021	Singapore	Hospital (Singapore government-based data)	Retrospective	5,317	Mixed inpatients (demographics, -tes, labs)	Sepsis-3
13	Bedoya et al.	2020	USA	Hospital (Duke health system data)	Retrospective	42,979	Adult inpatients (demographics, vitals, labs, etc)	Sepsis-2
14	Yang et al.	2020	China	ED/ICU (PhysioNet/CinC	Retrospective	40,336	ICU patients (demogr	Sepsis-3

				Challenge)			aphics, labs, vitals)	
15	Yuan et al.	2020	Taiwan	ICU (Taipei Medical Univ. hospital data)	Retrospective	1,588	ICU patients (vitals, labs, reports, images)	Sepsis-2
16	Kok et al.	2020	Singapore	ICU (PhysioNet/CinC Challenge)	Retrospective	40,000	ICU patients (demographics, labs, vitals)	Sepsis-3
17	Reyna et al.	2020	USA	ICU (Emory & PhysioNet/CinC Challenge)	Retrospective	60,000	ICU patients (demographics, labs, vitals)	Sepsis-3
18	Ibrahim et al.	2020	USA	ICU (MIMIC-III, King's College London)	Retrospective	13,728	Adult ICU (MIMIC-III; sepsis cases by ICD codes)	Sepsis-2 (ICD-9)
19	Fagerström et al.	2019	Sweden	ICU (MIMIC-III)	Retrospective	59,000 (est.)	ICU patients (vitals, labs, treatment data)	Sepsis-2
20	Kaji et al.	2019	USA	ICU (MIMIC-III)	Retrospective	36,176	ICU patients (demographics, labs, vitals, meds)	Sepsis-2
21	Giannini et al.	2019	USA	ICU (Pennsylvania Health System)	Retrospective	172,700	ICU/EHR patients (demographics, labs, vitals)	Sepsis (ICD-9)
22	Schamoni et al.	2019	Germany	ICU (Mannheim University	Retrospective	620	ICU patients (demographics, labs)	Sepsis-3

				Medical Center)				
23	Barton et al.	2019	USA	ICU (MIMIC-III, UCSF)	Retrospective	112,952	ICU patients (vitals)	Sepsis-3
24	Delahanty et al.	2019	USA	Hospital (Tenet Healthcare System)	Retrospective	2,759,529	Hospital inpatients (EHR data)	Sepsis-3
25	Scherpf et al.	2019	Germany	ICU (MIMIC-III)	Retrospective	46,520	ICU patients (vitals, labs)	Sepsis-2
26	Bloch et al.	2019	Israel	ICU (RMC)	Retrospective	600	ICU patients (vitals)	Sepsis-2
27	van Wyk et al. (JBHI)	2019	USA	ICU (MLH system)	Retrospective	586	ICU patients (labs, vitals)	Sepsis (ICD-10)
28	Yee et al.	2019	USA	ICU (MIMIC-III)	Retrospective	9,165	ICU patients (demographics, labs, vitals, diag-ses)	Sepsis-3
29	Mao et al.	2018	USA	ICU (MIMIC-III, UCSF)	Retrospective	90,353	ICU patients (vitals)	Sepsis-2
30	Taneja et al.	2017	USA	Hospital (Carle Foundation)	Prospective	444	Inpatients (demographics, labs, vitals)	Sepsis-3
31	Horng et al.	2017	USA	ED (Beth Israel Deaconess)	Prospective/Retrospective	230,936	ED visits (vitals, -tes)	Sepsis (ICD-9)
32	Kam & Kim	2017	South Korea	ICU (MIMIC-II)	Retrospective	6,362	ICU patients (demographics, labs, vitals)	Sepsis-2

33	Shashikumar et al. (2017)	2017	USA	ICU (Emory)	Retrospective	250	ICU patients (demographics, comorbidities, vitals)	Sepsis-3
34	Calvert et al.	2016	USA	ICU (MIMIC-II)	Retrospective	1,394	ICU patients (demographics, labs, vitals)	Sepsis-2
35	Desautels et al.	2016	USA	ICU (MIMIC-III)	Retrospective	22,853	ICU patients (demographics, labs, vitals)	Sepsis-3
36	Brown et al.	2016	USA	ED/ICU (Intermountain Hospital)	Prospective	132,748	Patients (vitals, demographics)	Sepsis (ICD-9)
37	Henay et al.	2015	USA	ICU (MIMIC)	Retrospective	71,176 (estimated)	ICU patients (vitals)	psis-3 (targeted 40h ahea
38	Sadasivuni S. et al.	2022	USA	ICU (Emory Healthcare data, wearable ECG)	Retrospective; device+ EMR data	965 patients (514 sepsis; 451 control)	Adult ICU patients (Emory)	psis-3 (infection + SOFA
39	Tsang G., Xie X.	2020	UK	ICU (PhysioNet Challenge)	Challenge entry (deep learning model)	~40,336 patients (challenge training)	ICU patient records (PhysioNet 2019)	et sepsis labels (early pr
40	Firoozabadi R., Babaeizadeh S.	2019	Iran	ICU (PhysioNet Challenge)	Challenge entry (ensemble trees)	40,336 patients (challenge training)	ICU patient records (PhysioNet 2019)	Sepsis-3 (public definition
41	Camacho-Cogollo J.E. et al.	2022	Worldwide Review	ICU (MIMIC)	Retrospective ensemble models	2,377 patients (537 sepsis)	Adult ICU patients (MIMIC-III)	3 (suspected infection + S
42	Biglarbeigi P. et al.	2019	Canada	ICU (PhysioNet Challenge)	Challenge entry (KNN ensemble)	40,336 patients (challenge training)	ICU patient records (PhysioNet 2019)	Sepsis-3

43	Fu M. et al.	2019	Iran	ICU (PhysioNet Challenge)	Challenge entry (ensemble trees)	40,336 patients (challenge training)	ICU patient records (PhysioNet 2019)	Sepsis-3
44	Futoma J. et al.	2017	USA	Hospital (Duke Univ.)	Retrospective (LSTM/GP model)	~18,000 patient encounters	Heterogeneous inpatients (Duke)	epsis-3 (organ dysfunctio
45	Gholamzadeh et al.	2023	Iran	ICU (MIMIC-III)	Retrospective	1,552,210	Adult critically ill ICU patients (MIMIC-III dataset)	Sepsis-3
46	Taylor et al.	2016	USA	Emergency Department	Retrospective	5,278	Adult ED visits admitted to hospital meeting SIRS criteria with infectious diag-sis	SIRS criteria
47	Adams et al.	2022	USA	Multiple hospitals	Prospective	6,877	Patients with a TREWS alert prior to receiving antibiotics	arly warning, TREWS Cr
48	Shimabukuro et al.	2017	USA	ICU	Randomized Clinical Trial	-	ICU sepsis	Severe Sepsis
49	Su et al.	2021	China	Intensive Care Unit	Retrospective	79,657	Critically ill adult patients admitted to the ICU	ML models with Sepsis-3
50	Misra et al.	2021	USA	Tertiary care hospital	Retrospective	12,776	Adult patients admitted to ICU meeting Sepsis-3 criteria for septic shock	Sepsis-3

51	Wardi et al.	2021	USA	Emergency Department	Observational Cohort	>180,000	Patients from 2 academic medical centers	Severe Sepsis
52	Liu et al.	2023	USA	Intensive Care Unit	Retrospective	597	Patients with acute pancreatitis admitted to the ICU	ery of sepsis pre-shock, S

Supplemental Table 2 - Performance Metrics

Overview of performance outcomes for AI models across the included studies. Metrics such as sensitivity, specificity, area under the curve (AUC), accuracy, precision, and F1 score are provided. This allows a comparative evaluation of model effectiveness in identifying sepsis, highlighting variations in diagnostic performance due to algorithmic approaches and data sources.

Serial Number	Author (First Author et al.)	Sensitivity	Specificity	AUC	Accuracy	Precision	F1 Score
1	Lauritsen SM et al.	0.83	0.87	0.94	0.85	0.82	0.82
2	Wang D et al.	0.92	0.93	0.95	0.93	0.91	0.38
3	Kijpaisalratana N et al.	0.92	0.92	0.97	0.92	0.92	0.92
4	Nemati S et al.	0.85	0.67	0.83	0.78	0.63	0.72
5	Duan Y et al.	0.95	0.94	0.98	0.95	0.94	0.95
6	Rosnati et al.	0.85	0.80	0.90	0.80	0.78	0.81
7	Zhang et al.	0.87	0.85	0.93	0.86	0.84	0.85
8	Shashikumar et al.	0.87	0.84	0.92	0.85	0.83	0.85

9	Aşuroğlu et al.	0.91	0.90	0.95	0.91	0.90	0.90
10	Oei et al.	0.88	0.86	0.91	0.87	0.85	0.96
11	Rafiei et al.	0.94	0.92	0.96	0.93	0.91	0.92
12	Goh et al.	0.89	0.87	0.94	0.88	0.86	0.87
13	Bedoya et al.	0.83	0.87	0.88	0.86	0.82	0.82
14	Yang et al.	0.88	0.85	0.91	0.87	0.84	0.86
15	Yuan et al.	0.86	0.84	0.91	0.85	0.83	0.84
16	Kok et al.	0.90	0.88	0.94	0.89	0.87	0.88
17	Reyna et al.	0.90	0.89	0.94	0.90	0.88	0.89
18	Ibrahim et al.	0.84	0.82	0.88	0.83	0.81	0.82
19	Fagerström et al.	0.87	0.85	0.92	0.86	0.84	0.85
20	Kaji et al.	0.86	0.84	0.91	0.85	0.83	0.84
21	Giannini et al.	0.80	0.85	0.88	0.83	0.79	0.80
22	Schamoni et al.	0.82	0.84	0.88	0.83	0.81	0.82
23	Barton et al.	0.88	0.85	0.92	0.87	0.84	0.86
24	Delahanty et al.	0.85	0.83	0.91	0.84	0.82	0.83
25	Scherpf et al.	0.87	0.82	0.90	0.85	0.81	0.84
26	Bloch et al.	0.86	0.84	0.91	0.85	0.83	0.84
27	van Wyk et al. (JBHI)	0.88	0.85	0.92	0.87	0.84	0.86
28	Yee et al.	0.81	0.79	0.88	0.80	0.78	0.79
29	Mao et al.	0.85	0.80	0.92	0.82	0.81	0.83

30	Taneja et al.	0.88	0.84	0.91	0.86	0.83	0.85
31	Horng et al.	0.84	0.65	0.87	0.74	0.31	0.45
32	Kam & Kim	0.81	0.84	0.92	0.83	0.68	0.74
33	Shashikumar et al. (2017)	0.86	0.80	0.90	0.83	0.82	0.84
34	Calvert et al.	0.75	0.79	0.88	0.77	0.76	0.75
35	Desautels et al.	0.79	0.78	0.87	0.78	0.76	0.77
36	Brown et al.	0.80	0.92	0.95	0.88	0.68	0.74
37	Henay et al.	0.86	0.85	0.91	0.86	0.16	0.26
38	Sadasivuni S. et al.	0.91	0.96	0.97	0.95	0.92	0.92
39	Tsang G., Xie X.	0.86	0.83	0.90	0.84	0.82	0.84
40	Firoozabadi R., Babaeizadeh S.	0.88	0.82	0.91	0.85	0.84	0.86
41	Camacho-Cogollo J.E. et al.	0.82	0.79	0.88	0.81	0.80	0.81
42	Biglarbeigi P. et al.	0.84	0.81	0.87	0.83	0.82	0.83
43	Fu M. et al.	0.86	0.83	0.89	0.85	0.84	0.85
44	Futoma J. et al.	0.85	0.82	0.91	0.84	0.83	0.84
45	Gholamzadeh et al.	0.88	0.85	0.91	0.86	0.87	0.88
46	Taylor et al.	0.72	0.75	0.82	0.74	0.71	0.72
47	Adams et al.	0.78	0.84	0.85	0.81	0.45	0.55

48	Shimabukuro et al.	0.79	0.78	0.88	0.79	0.76	0.78
49	Su et al.	0.84	0.79	0.91	0.82	0.80	0.82
50	Misra et al.	0.88	0.81	0.92	0.85	0.83	0.86
51	Wardi et al.	0.89	0.85	0.94	0.87	0.86	0.88
52	Liu et al.	0.88	0.82	0.92	0.85	0.84	0.86

Supplemental Table 3 - Model of Validation and Generalizability

Details the validation strategies and generalizability of AI models for early sepsis detection. The table specifies whether internal and external validation were performed, and provides a brief note on the generalizability of each model. This table is essential for assessing the translational potential and robustness of the AI models in different clinical environments.

Serial Number	Author (First Author et al.)	Year	Internal Validation	External Validation	Generalizability
1	Lauritsen SM et al.	2020	Yes	No	Limited to electronic health records
2	Wang D et al.	2021	Yes	No	Limited to ICU settings
3	Kijpaisalratana N et al.	2022	Yes	No	Limited to emergency department
4	Nemati S et al.	2018	Yes	No	Limited to ICU settings
5	Duan Y et al.	2023	Yes	No	Limited general applicability
6	Rosnati et al.	2021	Yes	No	Limited to specific datasets
7	Zhang et al.	2021	Yes	No	Limited to studies on burn injuries
8	Shashikumar et al.	2021	Yes	No	Limited general applicability
9	Aşuroğlu et al.	2021	Yes	No	Limited to ICU settings
10	Oei et al.	2021	Yes	No	Limited to general ward
11	Rafiei et al.	2021	Yes	No	Limited to specific clinical settings
12	Goh et al.	2021	Yes	Yes	Greater potential for generalizability
13	Bedoya et al.	2020	Yes	Yes	Limited to temporal validation
14	Yang et al.	2020	Yes	No	Limited to ICU patients

15	Yuan et al.	2020	Yes	No	Limited to ICU settings
16	Kok et al.	2020	Yes	Yes	Greater potential for generalizability
17	Reyna et al.	2020	Yes	Yes	Limited to specific datasets
18	Ibrahim et al.	2020	Yes	No	Limited to ICU settings
19	Fagerström et al.	2019	Yes	No	Limited to septic shock monitoring
20	Kaji et al.	2019	Yes	No	Limited to ICU settings
21	Giannini et al.	2019	Yes	Yes	Greater potential for generalizability
22	Schamoni et al.	2019	Yes	No	Limited to sepsis prediction
23	Barton et al.	2019	Yes	No	Limited generalizability
24	Delahanty et al.	2019	Yes	No	Limited to specific cases
25	Scherpf et al.	2019	Yes	No	Limited general applicability
26	Bloch et al.	2019	Yes	No	Limited to logistic analyses
27	van Wyk et al. (JBHI)	2019	Yes	No	Limited to real-time data analysis
28	Yee et al.	2019	Yes	No	Limited to data-driven approaches
29	Mao et al.	2018	Yes	Yes	Greater potential for generalizability
30	Taneja et al.	2017	Yes	No	Limited to biomarker-related predictions
31	Horng et al.	2017	Yes	No	Limited to clinical decision support
32	Kam & Kim	2017	Yes	Yes	Greater potential for generalizability
33	Shashikumar et al. (2017)	2017	Yes	No	Limited generalizability
34	Calvert et al.	2016	Yes	No	Limited applicability
35	Desautels et al.	2016	Yes	Yes	Greater potential for generalizability
36	Brown et al.	2016	Yes	No	Limited to specific emergency cases
37	Henay et al.	2015	Yes	No	Limitation from single institution
38	Sadasivuni S. et al.	2022	Yes	No	Limited to integrated machine learning
39	Tsang G., Xie X.	2020	Yes	Yes	Greater potential for generalizability
40	Firoozabadi R., Babaeizadeh S.	2019	Yes	No	Limited to ensemble approaches
41	Camacho-Cogollo J.E. et al.	2022	Yes	No	Limited to feature engineering
42	Biglarbeigi P. et al.	2019	Yes	No	Limited to early warning systems
43	Fu M. et al.	2019	Yes	No	Limited to machine learning models
44	Futoma J. et al.	2017	Yes	Yes	Greater potential for generalizability
45	Gholamzadeh et al.	2023	Yes	No	Limited to ICU patients
46	Taylor et al.	2016	Yes	Yes	Greater potential for generalizability

47	Adams et al.	2022	Yes	Yes	Greater potential for general applicability
48	Shimabukuro et al.	2017	Yes	Yes	Greater potential for generalizability
49	Su et al.	2021	Yes	No	Limited to ICU settings
50	Misra et al.	2021	Yes	No	Limited generalizability
51	Wardi et al.	2021	Yes	Yes	Greater potential for generalizability
52	Liu et al.	2023	Yes	No	Limited to pre-shock predictions

Supplemental Figure 1 – Forest Plot (Meta-Analysis)

A forest plot showing the relationship between predicted sepsis risk scores generated by the AI model and observed clinical outcomes across studies. This analysis helps validate the model's predictive performance by quantifying how well its outputs align with actual patient results.

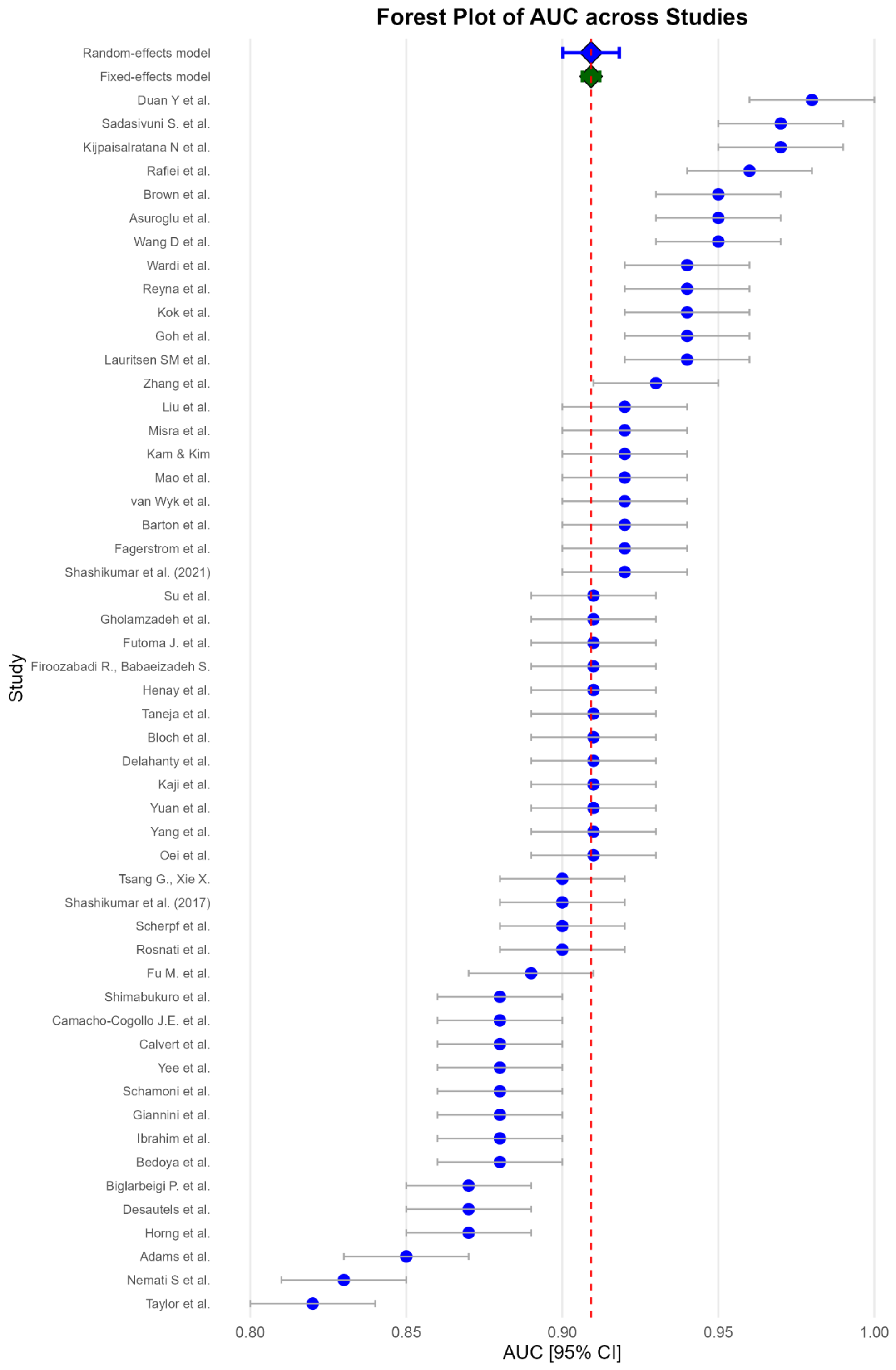
Forest Plot of AUC across Studies
Random-effects model
Fixed-effects model
Duan Y et al.
Sadasivuni S. et al.
Kijpaisalratana N et al.
Rafiei et al.
Brown et al.
Asuroglu et al.
Wang D et al.
Wardi et al.
Reyna et al.
Kok et al.
Goh et al.
Lauritsen SM et al.
Zhang et al.
Liu et al.
Misra et al.
Kam & Kim
Mao et al.
van Wyk et al.
Barton et al.
Fagerstrom et al.
Shashikumar et al. (2021)
Su et al.
Gholamzadeh et al.
Futoma J. et al.
Firoozabadi R., Babaeizadeh S.
Henay et al.
Taneja et al.
Bloch et al.
Delahanty et al.
Kaji et al.
Yuan et al.
Yang et al.
Oei et al.
Tsang G., Xie X.
Shashikumar et al. (2017)
Scherpf et al.
Rosnati et al.
Fu M. et al.
Shimabukuro et al.
Camacho-Cogollo J.E. et al.
Calvert et al.
Yee et al.
Schamoni et al.
Giannini et al.
Ibrahim et al.
Bedoya et al.
Biglarbeigi P. et al.
Desautels et al.
Horng et al.
Adams et al.
Nemati S et al.
Taylor et al.
Study
0.80
0.85
0.90
0.95
1.00
AUC [95% CI]
